# Crafting the Isolated Consulting Room: Practices of Independence and Collaboration of Finnish Primary Care GPs

**DOI:** 10.1111/1467-9566.70071

**Published:** 2025-07-23

**Authors:** Inka Laisi

**Affiliations:** ^1^ Centre of Excellence in Research on Ageing and Care RG3 Migration, Care and Ageing University of Helsinki Helsinki Finland

**Keywords:** crafting, general practitioner, job crafting, material‐discursive practices, primary healthcare, professional isolation

## Abstract

This study examines professional isolation in Finnish public primary healthcare and how general practitioners (GPs) craft their work in health centres and in the doctor's consulting room in particular. Consulting rooms are often places of isolation and loneliness but simultaneously arenas of independence and autonomy. The data consisted of 16 interviews of Finnish doctors, out of whom 13 worked in public health centres or were specialising in general practice at the time of the interview and three worked in hospitals. This study shows how institutional material‐discursive practices that create the consulting room isolate the GP and how this generates feelings of loneliness, as well as how GPs aim to make the workplace more inhabitable through crafting themselves as independent and autonomous doctors or through crafting the environment to enable collaborative relationships. Through this kind of crafting, the GPs create professionally sustainable jobs and roles for themselves in the public primary healthcare organisation.

## 1 Introduction

General practitioners (GPs) in Finland and other countries with developed healthcare systems commonly report experiencing feelings that researchers have interpreted as *professional isolation* (Aira et al. 2010; Larkins et al. 2004). Professional isolation concerns aspects of clinical work, such as physical separation from other professionals, making decisions alone or the lack of senior support, opportunities to consult on medically difficult cases or for debriefing (ibid.). Finnish studies have found that professional isolation causes emotional distress and feelings of loneliness on top of the aforementioned problems, ultimately driving doctors away from primary healthcare (Lepäntalo et al. 2008). GPs' work is emotionally burdensome even without isolation. The vulnerability and emotional burden of medical doctors is a well‐researched topic (e.g., Gothill and Armstrong 1999; Litvina et al. 2020; Nettleton et al. 2008) and further builds the knowledge we have about this professional group with relatively great societal power and authority.

This article examines professional isolation in a Finnish public primary healthcare organisation, the health centre and in the GP's consulting room, in particular. The research questions are as follows: How does professional isolation occur in Finnish health centres? What do the GPs do in response? The article's contribution is twofold. First, it shows how institutional *material‐discursive practices* (Barad 2007; Orlikowski and Scott 2015) of the consulting room isolate the GP, and second, how GPs *craft* either themselves as independent or their surroundings as collaborative by enacting different materially, discursively and socially embedded practices.

In approaching professional isolation, my starting point is in institutionalist and organisational theory, and I aim to analyse how the institutional role that doctors inhabit in the health centre is sociomaterially organised. The sociomaterial perspective entails an interest in the concrete environment of primary healthcare work: the GP's consulting room. The sociomaterial or *material‐discursive* (originally conceptualised by Karen Barad) nature of these practices means that discourses and the material are seen as ontologically inseparable (Barad 2007; Orlikowski and Scott 2015, 2023). Through practices, the world and ‘reality’ are constantly remade, and ‘for anything to exist, it must be materialised in specific times and places through actions, texts, models, codes, and so on’ (Orlikowski and Scott 2023, 6). Thus, the material consulting room does not exist without its discursively given meanings and vice versa; the discourses framing GPs’ work do not exist without being materialised in spaces. I will show how professional isolation in the consulting room is a result of different sociomaterial practices that isolate the doctor and shift the focus from a static and fixed concept of professional isolation found in previous studies (Aira et al. 2010; Larkins et al. 2004) to a constantly changing process involving multiple concurrent practices.

To emphasise doctors' roles as not only ‘inhabitants’ of the organisation they work in but also as crafters of their daily work and work trajectories, I analyse how professionals manage ambiguous demands and make sense of the problems faced at work. *Crafting* in the context of medical work and care has many meanings and has been the subject of many theoretical discussions. *Job crafting* is a concept developed by organisational psychologists Wrzesniewski and Dutton (2001) to describe actions such as changing the number, scope and type of job tasks; changing the quality and/or the amount of interaction with others or changing cognitive task boundaries in order to redefine and shape work. By crafting their jobs, workers aim to create better work trajectories for themselves, improve job satisfaction and enhance work identity or meaning (Wrzesniewski and Dutton 2001). For example, Fuller and Unwin (2017) have shown how people doing ‘dirty work’ change the primary function of their work to retain high esteem and pride, and Yu and Jyawali (2021) have shown how women adapt to the masculine work environment by negotiating competence and reframing creativity. In a recent study on nurses, Felder et al. (2024) show how job crafting can be a valuable retention strategy but is still restricted by the policies and conventions of medical organisations. Carmel (2013) has used the concept of craft in analysing the application of different kinds of knowledge and the interaction with the materials in medical work, offering a broader understanding of medical work than the traditional science/art dichotomy. Crafting relates also to the concept of *tinkering* (Mol et al. 2010; Wallenburg et al. 2013), which refers to incorporating different, sometimes conflicting, practices together in order to make things work out. My perspective on crafting focuses on how GPs craft their workspaces, roles and relationships, that is, the organisation, to make sense of the ‘reality’ of their work that is produced through material‐discursive practices. By looking at crafting in the sociomaterial context of the consulting room, I aim to understand how human actors—GPs—make sense of their work while being a part of the practices themselves.

## 2 The Material‐Discursive Consulting Room

Scholars have called for a ‘place‐sensitive’ sociology (Gieryn 2000) and a thorough examination of material elements and physical infrastructures in the context of health, illness and care (Ivanova et al. 2016; Martin et al. 2015; Nettleton et al. 2018). Here, the interest lies in the material‐discursive practices creating the doctor's consulting room. The room has many sociological interpretations that describe the relationship between the doctor and the patient: It is the stage (Goffman 1956) at which the doctor and patient meet, an encounter in which the voice of the lifeworld comes across the voice of medicine (Mishler 1984) or a spatial territory for control and power (Eräsaari 1995). Architecturally, consulting rooms adapt to a wider style of public office and bureaucracy space design which—before open‐office design—resembled the Foucauldian ‘prison cell system’ and the ‘strategy of individualism’, which Eräsaari (1995, 137–48) has recognised in the Finnish context as well in her classic research on bureaucratic spaces. Although industrial offices with their ‘cubicles’ and visible hierarchies have been reformed into post‐industrial open offices in many sectors, in the health centre the cubicle prevails, presumably for patient privacy and information security reasons. Eräsaari (1995, 148) describes how the dominance of the ‘cell office system’ links to Finnish culture in general: ‘It is an architecture of loneliness, silence, sensitivity, shyness and anxiety’ (author's translation). Thus, the physical space serves as a site of cultural understandings (Gieryn 2000).

The health centre is an organisation with formal ideals and rationales, informal work practices, social interactions and physical infrastructures. Whereas old institutionalism sees doctors as constructing the health centre through their medical and organisational work, new institutionalism sees doctors as the result of the centre's organising logics and norms. Inhabited institutionalism (II) is somewhere in between these and suggests examining the institutions locally, focusing on how interaction, organisation and institutional ideals are related to each other (Hallett and Hawbaker 2021). ‘II is at its best in examining ‐ ‐ how the meanings and implications of institutional myths and rationales get worked out in social interactions, and how those meanings become the basis of ongoing organisational activities which have implications for the very institutions that shape organisations and the people who enliven them’ (Hallett and Hawbaker 2021, 21). VanHeuvelen (2019) complements II by zooming in on the built environment and how it shapes the way in which people work. VanHeuvelen (2019) showed how the spatial change in the design of a children's intensive care unit facility affected the local work culture by limiting opportunities to meet and communicate with other workers, producing feelings of isolation and increasing individual responsibility in care assessment. However, employees maintained the collaborative culture by inventing new ways of meeting and communicating (VanHeuvelen 2019).

As stated above, many scholars see the discursive and the material reality being intertwined when examining the use of spaces. However, as Orlikowski and Scott (2015, 2023) argue, leaning on Barad’s (2007) agential realism, discourse and material are ontologically inseparable. Thus, their focus is on *practices* that are material‐discursive (or sociomaterial) and constantly constitute the world. Orlikowski and Scott (2015, 703) encourage organisation and management scholars to move from studying interaction between entities to ‘concern ourselves with detailing how specific materialisations of discourse make a difference in practice, and with what performative consequences’. Therefore, the organisational logics cannot be separated from the physical setting, nor can interactional habits be separated from the places where people work. Building on these perspectives, I will show how both phenomena, professional isolation and GPs' ways of crafting their work, are entangled with the physical spaces, organisational logics and norms, professional discourses and interaction between different (human and nonhuman) actors.

## 3 GPs in Public Primary Care in Finland

GPs' work includes many tensions ranging from the individual level to the historical professional level. GPs are considered one of the least prestigious groups among medical specialities, which has been explained by, for example, the close relationship with patients and the general medical knowledge base (Norredam and Album 2007). As general practice deals with older, multimorbid patients and involves less technology and more social problems, it is positioned lower, and thus, work in health centres can be considered ‘dirty work’ (Hughes 1984) within the medical profession. According to a recent study, GPs in Finland feel less appreciated by colleagues from other specialities than hospital doctors (Wrede et al. 2022). However, many international studies have described general practice as one of the ‘lifestyle friendly’ specialities (Creed et al. 2010) and as ‘nice work’ (Jones and Green 2006).

The institutional position of most Finnish GPs is that of an employee in a public health centre, which originates from the Public Health Care Act of 1972 and which created a locally anchored yet nationally steered primary health service for Finnish citizens. Primary healthcare services collect client charges, but services are mostly free of charge. Currently, the primary health service consists of 138 health centres and around 4000 GPs (The Finnish Medical Association 2022). Until the social and healthcare reform that shifted the organising role to the newly established welfare services counties in January 2023, primary care was organised by municipalities, and health centres' systems of organising medical care varied significantly. In some health centres, doctors were assigned a pool of patients in accordance with the ‘population responsibility model’ or the ‘personal doctor’ model. These population groups were often 2000–3000 people per doctor and usually composed based on residential areas. This model was often critiqued for its uneven distribution of health problems, although it also enabled some kind of continuity of care. The other prevalent model was a ‘working‐hour model’ in which doctors worked according to weekly schedules and patients saw the doctor that was available. In this model, the doctors work overtime less often, but continuity of care is endangered. In Finland, the working population has separate occupational healthcare, which is mainly organised by private service providers and paid by the employers. Thus, primary care patients are mostly older people, unemployed, children or other nonworking people. This dual healthcare model is one significant factor in increasing and maintaining health inequalities among Finnish people (Manderbacka et al. 2017).

Currently, the financial situation of welfare counties is alarming, and the Finnish government has responded to the situation by, for example, introducing cuts in services, increasing compensations for private health services and bringing back the so‐called ‘personal doctor’ model to ensure the continuity of care. Thus, the primary care sector is undergoing dramatic challenges and changes that affect the professionals working in these organisations. The labour shortages and heavy workloads of health centre GPs are much debated. Doctors' professional associations maintain that the primary health service suffers from long‐term resource problems, which are reflected in problems in recruiting medical personnel. The Finnish Medical Association (2023) has reported a lack of 300 GP vacancies in public health centres (8% of all GP positions) and that as many as 200 new positions would be required to meet the need for primary medical care.

On an average day in the health centre, GPs meet two to four patients in an hour, give consultations and do reporting and documentation work, such as writing certificates for patients. Although specialised GPs are (or should be) the primary group of doctors in health centres, there are actually six groups of doctors: medical students in temporary positions, recently graduated doctors carrying out their obligatory 9‐month primary care period (a political decision to ensure there are doctors in primary care), GPs specialising in general practice, already specialised GPs, nonspecialised GPs who have a long career in primary care and doctors with other specialist degrees than general practice. This has various effects depending on the stability and continuity of the workforce. Early‐career doctors need experienced mentors, but in rural areas where recruiting permanent staff is difficult, health centres might rely heavily on recently graduated doctors. A survey carried out by the Finnish Junior Doctors' Association (2021), in turn, found that 44% of junior doctors lacked mentoring in their place of speciality training.

## 4 Methods and Data

The data used in this study are a selection of 16 interviews from a larger set (*N* = 38) that were collected in 2016 for a research project on doctors' autonomy and professionalism. The interviews include both junior and senior as well as male and female doctors who were recruited via the Finnish Medical Association. Junior refers to doctors who graduated in 2010–2014, and senior refers to doctors over 60 years of age with 20 or more years of experience in medicine. The doctors worked in the capital city area (Helsinki) or a smaller city area (Tampere), and the workplaces varied in the number of colleagues and in organisational practices. Out of the 16 interviews (see Table 1), 13 doctors worked in health centres, were specialised GPs or were specialising in general practice at the time of the interview, and three worked in hospitals. I have included three hospital doctors in the analysis because they compared their work to GPs working in health centres and described differences in the amount of teamwork. Professional isolation can occur in all kinds of medical consulting tasks where the work is conducted mostly alone, so also in the private sector, but together with other current challenges in public primary healthcare, the main research interest is in GPs' work in health centres.

**TABLE 1:** Selected interviews (*N* = 16).

Number of interview	Career phase	Gender	Work place	Code
1	Senior	Female	Health centre GP	1SF
4	Junior	Female	Hospital	4JFH
7	Senior	Female	Health centre GP	7SF
8	Junior	Female	Health centre GP	8JF
10	Junior	Male	Hospital	10JMH
11	Junior	Female	Private sector GP	11JFP
13	Senior	Female	Health centre GP	13SF
15	Junior	Female	Health centre GP	15JF
23	Senior	Female	Hospital	23SFH
24	Junior	Male	Health centre GP	24JM
27	Senior	Male	Private sector GP	27SMP
34	Junior	Male	Health centre GP	34JM
35	Junior	Male	Health centre GP	35JM
36	Junior	Male	Health centre GP	36JM
37	Senior	Male	Health centre GP	37SM
38	Junior	Male	Health centre GP	38JM

The semi‐structured thematic interviews were conducted jointly by two research assistants in Finnish, and they lasted from 40 min to 2 hours. Although the interviewers asked the participants the same questions in the same order, they had been instructed to also pose some clarifying questions. The interview covered demands and threats at work, relationships with colleagues and patients, ideas about autonomy and the position of doctors in society. The doctors were given vignettes and statements about clinical situations (see Supporting Information S1: Appendix 1), on which they were asked to reflect and tell the interviewer whether they agreed or disagreed.

I was not involved in the data gathering nor in the original research project. Thus, I am using the interviews as secondary data, with the permission of the primary investigator of the project. In using secondary data, it is important to evaluate methodological rigour and ethical issues (Ruggiano and Perry 2017). Using secondary data is challenging because the interviews were not collected with the purpose of this study in mind. Professional isolation was not explicitly investigated in the interviews, but when asked to describe their workday, work community, benefits or challenges in working with others or different attitudes towards work, for example, between hospital and health centre, men and women or junior and senior doctors, the GPs began talking about isolation and loneliness. The interviewed doctors could have avoided bringing up the topic, but they chose to talk about their experiences, which highlights the importance of analysing the topic. Risks in using secondary data include ethical and methodological concerns, such as informed consent, confidentiality and changes in the cultural, social or political context where the data were originally collected (Ruggiano and Perry 2017). I have tried to avoid these by conducting systematic coding and by comparing findings with the parent study. Additionally, GPs are already burdened by their work, which makes using secondary data ethically more sustainable than collecting new data. Furthermore, this study adheres to the ethical standards set out by the Finnish National Board on Research Integrity (TENK). According to guidelines defined by TENK, an ethical review is only required in specific ethically challenging scenarios, none of which apply to this study. The data that support the findings of this study are not available due to privacy or ethical restrictions.

I conducted the analysis by combining methods of grounded theory (Timonen et al. 2018) and abductive content analysis (Earl Rinehart 2021). I noticed how GPs described their consulting rooms by using material metaphors to describe the problematic features of work, such as busy schedules and the lack of colleagues. I decided to code the strenuous working conditions and difficulties that the GPs faced when trying to manage their work and found out how material structures emerged as impressions to describe the problems. Rooms, doors and tables were used to describe the time pressure in the health centre. For example, one interviewee says that *‘lunch tables are not even for eating lunch’* (24JM). The movement of actors or ideas happened through connecting doors between the consulting rooms, but doctors were often the ones who stayed still, stuck and isolated, whereas patients and nurses moved in and out. At the same time, the doctors described spatial dimensions that restricted them in practice, as well as professional, more abstract ideals that they found empowering: They described collegiality as a ‘*support pillar’* (15JF). Thus, the chosen discursive elements were closely attached to the material environment. According to Fairclough (2010, 351–55), stable and durable ‘order of discourse’ preserves and maintains certain ways of action and practice, and by using discourses, actors participate in maintaining the order. However, it is often not a deliberate choice, and actors may even use different discourses in the same sentence, some of which can also contradict each other. I perceive GPs' talk as a ‘bricolage’ (Fairclough 2010, 353) of different discourses, which build upon the material setting of the work and describe the practices that create the ‘lonely consulting room’. The dimensions of professional isolation (Aira et al. 2010) and sociology of places and materiality complemented the analysis, in which I continued to search for the doctors' actions.

## 5 Isolating Practices Creating the ‘Lonely’ Consulting Room

GPs mostly see patients alone in consulting rooms. Their work schedules often follow a certain routine, which is described as ‘*paperwork*, *calls and clinical work*. *Three sections every day*’ (7SF). Some GPs might work a few days a week in a different ward or unit, such as in schools or polyclinics, but most of the time, GPs work in designated consulting rooms in health centres.

We don’t have any coffee breaks. And on breaks you could meet; we’re on two floors so some people are here on the second floor and others on the first floor. So, we never meet colleagues. Sometimes it comes up that someone is away, because their patients come or they’ve been transferred, that’s very bad. Of course, I have very good relationships with the administration and nurses, so in the mornings I try to be, I come to work early so I can see the situation [talking about how she was a substitute chief physician when this routine started]. But if someone does not attend meetings, or does not come to the so‐called lunch break, then we may not meet colleagues for weeks. It’s so lonely, this work.

(7SF)

The interviewed senior GP describes how physical separation, lacking information and not being able to meet colleagues create loneliness. ‘*The so‐called lunch break*’ implies dissatisfaction, which she tries to avoid by building relationships with the people she is able to meet: the administrative staff and nurses. The previously reported professional isolation—the lack of senior help, making decisions alone, lack of collaboration and not being part of the community (Aira et al. 2010)—emerged in the empirical data. Many of the interviewed GPs use the word ‘lonely’ when describing their work, and the opportunities for collaboration and teamwork seem restricted due to the constant time pressure and spatial solutions that separated doctors from other workers. Isolation in health centres is often presented as opposite to the work in hospitals.

Q Are there any differences between attitudes to work in health centres, hospitals, and the private sector?

A ‐ ‐ Well, working in a health centre might be a bit lonelier than in a hospital, somehow, it can be, that in hospital you get, you're constantly surrounded by colleagues and nurses and other occupations and you get to do a lot of teamwork with others, the work of a general practitioner is more a kind of lonely grind in your own room, people come in and go out and come in and go out so, I don't know how to answer that.

(36JM)

The interviewed hospital doctors also recognise this difference:

A Yeah, the way of working is very different in the hospital and in the health centre. If you work only in your consulting room in the health centre, it's quite different. It's a bit more, like lonely work. Well, of course you have a nurse partner and there are meetings, and stuff. Here we start every morning with the X‐ray meeting, and everybody comes there, [‐‐] The work in the hospital is more like teamwork.

(10JMH)

In hospitals, doctors are often surrounded by colleagues and other staff members, but in health centres, doctors are alone in their consulting rooms. In the penultimate quote, the GP describes how other people, presumably nurses and patients, move in and out of the room, whereas he has to stay there. The feeling of being stuck refers to isolation (also, VanHeuvelen 2019, 701) and can reinforce the feeling of losing control over work. Quite a few interviewees use the Finnish word ‘*koppi’* in describing their consulting room. The word refers to a kennel or a shed and is used to describe office cubicles in a negative light. Being trapped or stuck in the room is not only practically problematic, but it can also have a symbolic meaning: My work is not valued as I'm left alone to survive. Research on Finnish GPs' career paths suggests that health centres are professional peripheries as opposed to the more prestigious hospitals (Wrede et al. 2022).

But then if you think hospital versus health centre, so very few have enjoyed working in health centres. It’s often the feeling of uncertainty that is more common here, the lack of resources, the constant time pressure, that you don’t necessarily have the support that you often do in specialised healthcare [i.e. hospitals]. In practice, lunch tables are not even for eating lunch. So, health centres are often a scary thing for young doctors because you’re alone there.

(24JM)

The fact that ‘*lunch tables are not even for eating lunch’* suggests that health centres are chaotic, the workers do not have enough time to execute necessary tasks or have any breaks, or that the materials at hand are not the most relevant or useful. All of this creates uncertainty. In the data, doctors describe more the physical infrastructures that create problems and isolate them from others rather than explicitly talk about lacking significant colleagues or help in clinical decisions. The health centre becomes a lonely and frightening place full of uncertainties and complexities. The descriptions of the health centre as a scary place and the ‘*lonely grind’* (36JM) in the consulting room, in particular, show how the material and discursive are mutually constitutive, giving the work meaning, which is present in both the physical setting and the talk of the GPs. Thus, professional isolation occurs through material‐discursive practices.

Other doctors working mainly in consulting rooms, for example, in the private sector, might also experience isolation and loneliness, but together with other issues faced in public healthcare as well as the GP deficit, GPs' isolation is worrying. These situations mobilise negative emotions. Researchers elsewhere have identified positions such as the vulnerable (Litvina et al. 2020) and the feeling doctor (Nettleton et al. 2008) when analysing the emotional aspect of doctoring. Besides experiencing emotional loneliness, work in health centres seems to involve situations in which collegial and senior support is limited and making decisions alone becomes the norm. If the early career stage is full of isolation, loneliness or uncertainty, this can violate the psychological contract of career expectations in a way that threatens the production of sustainable medical careers (Mitra et al. 2023). Interestingly, early‐career hospital doctors recognise and talk about how much of the learning—becoming a doctor—happens through working with others:

A benefit in working with colleagues is that you learn a lot, especially when you are starting your career, and actually this is a kind of master‐apprentice profession, so a lot of the learning happens while working.

(4JFH)

The isolated consulting room is a result of institutional practices that can have many negative consequences for the doctors. Next, I will explore how the doctors make sense of their work and reframe loneliness to independence or collaboration.

## 6 Crafting the GP Job

In relation to the lonely consulting room, doctors participate in material‐discursive practices that challenge isolation and loneliness. Traditional job crafting practices such as planning one's own schedule and deciding on the order of tasks (Wrzesniewski and Dutton 2001) are used, but the opportunities to do so depend on the organisational model. A GP with a population responsibility has to take care of all their patients (even if the official working hours are not sufficient for this) but has more leverage in planning the schedule. GPs with a working‐hour model do not have to work overtime but are more restricted in planning their schedules and organising tasks. However, doctors describe the health centre as a more flexible work environment than the hospital because they mostly work office hours, have no on‐call responsibilities and have more control over schedules. The downside of flexibility, which usually correlates with underpaid and undervalued work, is having to deal with the burden of work individually rather than the institution being responsible for changing burdensome practices (Wrede et al. 2021). As crafters, GPs strive to change something in their work, and next, I show how they craft themselves as independent doctors within the boundaries of ‘good enough’ medical practice. The last section focuses on relations and collaboration and how spaces and materials need to be crafted to make collaboration possible.

### 6.1 Independence and Autonomy

Loneliness is usually defined as lacking significant relationships (e.g., de Jong Gierveld et al. 2006), and it is one aspect of professional isolation according to previous research (Larkins et al. 2004). However, people can be isolated and not feel lonely or be surrounded by people and in desperate need of social and emotional connection. Although GPs describe doing the work alone, they consider isolation and independence as two sides of the same coin.

Especially in the health centre, and consulting in particular, you do the work independently and alone.

(35JM)

This is kind of independent, lonely work here in the cubicle, you see patients, but you might not see colleagues except during lunch breaks.

(15JF)

Again, the doctors are stuck in the cubicle, but now they frame the work as independent, which seems like a proper discursive strategy to tackle the loneliness of the consulting room. The interviewed doctors link independence to clinical autonomy and making decisions.

Q How do you understand the idea of a doctor's autonomy?

A It's still the doctor who makes those decisions so you're alone in this room with the patients and at that moment there's no EBM [evidence‐based medicine] or colleagues or specialists, so you make the plan from there onwards. So, after all, you do the work yourself, at least here. In the hospital world there might be more group conversations and teamwork, but the attending doctor is still the one who makes the decisions.

(15JF)

Clinical autonomy is the cornerstone of medical professionalism (Freidson 1970) and might thus explain this discursive choice. In hospitals, decisions are pondered collectively, but ultimately, the attending doctor has the last word. Often in the health centre, the GPs ponder and make decisions mostly alone. Specific institutional material‐discursive practices influence the experience of work management. For example, the patient population responsibility model might enable greater autonomy than the working‐hour or multiprofessional team model but often means many overtime hours, as the next interviewee describes.

But, yeah of course, the fact that we don’t have office hours here because we have this population responsibility system, it kind of erodes your ability to control, like you could sit here for as long you can, but you need to know that you should go home. In the beginning I did a lot of overwork, it doesn’t show anywhere, you don’t get any extra compensation, as it’s kind of included in this, but my salvation was that we have a dog so [laughs]. Someone has to go and walk it, so you have something like that, and colleagues who have children, of course they have to, they finish at some point and if they can’t do everything in that time, then they do it the next day, mostly the paperwork, we definitely check all the patients. But you have to set the boundaries yourself.

(15JF)

In addition to highlighting independence in work management, the doctors seem to avoid describing the negative aspects of their work or downplay them by laughing, as in the previous quote. If you are expected to clear the list of patients, the only possible option to leave work is another, even more pressing duty, such as your own child or dog. However, early‐career and female doctors are often blamed for prioritising their personal lives and neglecting professional duties, such as working on call (Nettleton et al. 2008; Olakivi and Wrede 2019). For GPs, prioritising tasks seems like a crucial sign of professionalism; an independent, autonomous doctor knows when things are done ‘well enough’.

Of course you can use the whole 24 hours if you want to do everything perfectly. But that you do it well enough, everyone needs to figure that out themselves, the limit, when you’ve done things well enough.

(8JF)

Although work in consulting rooms might enable flexibility and independence, the reality of the health centre is also about constant time pressures, lack of resources and a fast pace (Markkanen et al. 2023). Limiting the amount of work is perceived as an individual responsibility, which highlights the individual endurance of the traditionally masculine and middle‐class profession (Olakivi and Wrede 2019). The interviewed doctors relied heavily on themselves—they craft themselves as independent in order to adapt to organisational demands. Recognising what is ‘good enough’ for the patients becomes an inherent aspect of GPs' work. VanHeuvelen's (2019) study also showed how isolated work increases the need to trust individual assessment skills, as more experienced workers were not that easily reachable. Performing independent and autonomous doctoring in isolated consulting rooms is a way to keep up a prestigious and professionally competent role. GPs avoid the normative and negative loneliness by claiming the space for independent doctoring and suppressing negative emotional responses.

### 6.2 Collaboration and Collegiality

Contrary to independence, GPs also emphasise effective collaboration, which helps make the days go by smoothly. Institutional material‐discursive practices, such as multiprofessional teams and teamwork with a nurse, are often used to secure collaboration opportunities. However, they cannot be all‐encompassing: unforeseen events, acute problems and delays need constant assessment and action—crafting—on the part of the employees. Thus, GPs actively craft their jobs by building relationships with others, creating new communication practices, finding places for consultation and moulding the material environment to better suit these purposes. In the following excerpt, the GP describes a flexible, effective work culture in which little things serve the big picture. Passing notes, being close to other staff members and organising tasks effectively make busy days go well.

We have a nurse partner model, so from my room I have access to the nurse’s room and we do, we work closely together, so it’s easy to delegate stuff to one another. And, it’s been really good with my nurse partner, and it’s actually expanded these hallways and we are a team, one line is a team here, and then there is a health nurse in the team as well. ‐ ‐ So, we can use everyone’s skills and competence. My nurse partner and I pass these little notes here and there, like can you check this and can you call here. Then often, if a patient comes and shows us some skin alterations, we often arrange it that they don’t have to book an appointment, they come to the nurse and then I check it. ‐ ‐ It takes, like, three minutes.

(15JF)

The spatial closeness to the nurse partner and the connecting door between their rooms is crucial for working together. ‘*Passing little notes’* is presumably not the organisation's official practice but a creative crafting practice developed in a specific context by the employees themselves. The interviewee describes how all the staff members could use their skills and competence to make the days pass smoothly—at least in the eyes of the doctor. The collaboration between a GP and a nurse is often about articulation work and dividing tasks so that they follow the positions of the medical hierarchy (Strauss et al. 1997). The effectiveness described above might look different from the nurse's perspective. Similarly, doctors often think that working with nurses includes the risk of being interrupted, which generates dissatisfaction (Lämsä et al. 2016). With a peer doctor, consulting in medically difficult cases is possible.

Q Are there any plusses to working with others?

A Yes. It's lovely, because if… Or like if you had to sit in this cubicle by yourself, it would be horrible [laughs]. It's nice when you have many workmates, and you can always share thoughts. And when you go to the coffee room, there's always somebody to chat with. Or when the connecting door [to another doctor's office] is open, we can share our thoughts or offload about why this is not working or what should I do with this. And that kind of thing. And catch up. It's a very big, important part of work.

(8JF)

The connecting door is open when the doctors do paperwork, which is usually described as the most time‐consuming task. It is important for them to ponder difficulties together and offer peer support. Otherwise, the work would be ‘*horrible*’. Having the door open and building supportive peer relationships seems to be a rewarding crafting practice and enables asking for help in a professionally sustainable way. Working together can also generate a feeling of having control over the huge workload. It produces a good team atmosphere, as one senior GP commented:

So you know you’re not doing this kind of work alone, like we do, we take care of this health centre, we care for the people of this area. In this specific area, they are our people, and we do this, we care for them together.

(1SF)

It is doubtful that this romantic view of ‘*taking care of our people*’ works for all GPs in all health centres. However, connecting doors and coffee rooms are material elements that help build a sense of connectedness and communality and enable resisting organisational power structures (Peteri et al. 2021). The connecting door means that the doctor stays in the consulting room, but the two separate rooms become a shared workspace where ideas flow and support is offered and received. Although GPs must open their doors to their patients, opening it to colleagues offers a different connection. Tolerating uncertainty becomes easier with a peer or with the help of a senior doctor, and to enjoy the independence of the job, the door can always be closed again. If independence was linked to clinical autonomy, collaboration is often connected to collegiality.

But, I would say that it [collegiality] is kind of a support in the background, like in the bigger picture, and the chance to consult with a specialist, and then at the workplace, as a closer and tighter support network. We know each other pretty well here, we spend some time together outside of work as well, so it’s easy to be in contact. Maybe the most important thing is that supporting pillar. ‐ ‐ so that you know you can ask for support and acceptance and help for decisions, even though you do it as if you were alone.

(15JF)

The discourse of collaboration and collegiality materialises in hallways, doors, coffee rooms and ultimately in the shared consulting room. Through crafting that promotes collaboration, GPs can work in an isolated room and still feel some connectedness and support. In contrast to crafting oneself as an independent doctor, here GPs focus on crafting their surroundings to enable collaborative relationships. Through these practices, the reality of the consulting room and health centre is constantly remade.

## 7 Discussion and Conclusions

Professional isolation and loneliness are usual and prevailing ways in which to frame GPs' work setting, not only in the data of this study but also in previous research (Aira et al. 2010; Larkins et al. 2004; Lepäntalo et al. 2008). My analysis confirms the importance of recognising professional isolation, such as loneliness, separation from other professionals and making decisions alone, in GPs' work, but I also argue that to understand the phenomenon, it is important to analyse the institutional material‐discursive practices that produce the ‘lonely consulting room’. According to the data, isolation occurred as a combination of physical separation, busy schedules and the lack of resources, which together left the GP stuck in the consulting room. As stated by the interviewed hospital doctors, this does not happen in the hospital environment. The lonely room reflects on the previously recognised positions of the vulnerable (Gothill and Armstrong 1999; Litvina et al. 2020) and feeling (Nettleton et al. 2008) doctor, who suffers from the negative aspects of isolation and loneliness. However, my analysis shows how loneliness is a result of practices and shifts the focus from the individual to the assemblage of materials, actors and relations. This highlights the fact that human action is never separate from spaces or materials and how ‘action’ is actually a result of material‐discursive practices. I argue that looking at material‐discursive practices advances inhabited institutionalism presented by Hallett and Hawbaker (2021) by showing how interaction, or the lack thereof, happens between people, things and places. Institutional change includes materials, discourses and actors that are entangled in practices.

For the interviewed GPs, human interaction matters. As previous research has shown, team job crafting and good team atmosphere generate positive outcomes for workers (Tims et al. 2013). Receiving and offering peer support turns the lonely cubicle into a shared place of thinking. Collaboration with a peer doctor was perceived as especially important; asking for help from a peer is probably the best way to maintain the role of a competent professional. Much research has shown that collaborative practices, such as peer or group support teams (Bedward and Daniels 2005), shared leadership (Aizenberg and Oplatka 2019) and Balint groups (Frey 2020), work effectively in overcoming professional isolation. The interviewed hospital doctors described their work as more collaborative compared to GPs working in consulting rooms in health centres. In the health centre, collaborative crafting practices happen with and through materials when workers pass notes, visit other spaces, such as coffee rooms, and keep doors open. Flexible, effective teamwork with a nurse improves articulation work (Strauss et al. 1997), which keeps the system going. Thus, collaboration with nurses helps doctors to control their work. Articulation work is an important part of professional and workplace autonomy (VanHeuvelen 2020), which highlights the importance of studying workplace practices that constantly constitute the reality of the organisation. Furthermore, collaboration is not the only strategy making isolated work more pleasant for Finnish GPs: Sometimes underlining independence and clinical autonomy works in reframing isolation.

Calling lonely work independent is a crafting practice through which doctors aim to create a more positive and professionally sustainable role for themselves. Although doctors talk about loneliness and recognise the problems caused by isolation, they simultaneously see it as an inherent part of the GP's work. Because of the GP deficit and organisational demands of efficiency, isolating practices can be seen as intrinsic in health centre work, aiming at producing ‘independent doctors’. The independent doctor, who is discursively explained with clinical autonomy, is expected to cope with stress, time pressure and difficult cases by themselves. They should know what to do and what is ‘good enough’. One acceptable reason for leaving work is another living being, such as a dog or a child that needs care. Sometimes GPs distance themselves from loneliness or downplay it, presumably because loneliness is perceived as stigmatising (Barreto et al. 2022). Independence and collaboration are crafting practices that GPs use simultaneously; sometimes enhancing independence might work better than emphasising relationships and vice versa. All interviewed doctors who talked about isolation also described their work as independent or their actions as collaborative. The same doctors can choose either one of the crafting strategies.

Wrzesniewski and Dutton (2001) originally acknowledged that being able to craft one's job is restricted by structural constraints, but much of the research has focused on capable individuals who overcome obstacles, mobilise resources and generate surpluses for organisations. Some critical research exists. For example, Felder et al. (2024) show how nurses create new roles for themselves through job crafting, but their actions are still restricted by human resource policies and traditional nursing conventions. They conclude that healthcare organisations should also improve nurses' political positions (ibid.). Here I have analysed crafting practices and shown how the ‘independent doctor’ is a result of crafting oneself to better suit the structural constraints of the job. However, crafting does not end there. This study acknowledges the mutual constitution of the material and the discursive by looking at the practices that create meanings and places. Lonely rooms are the result of institutional material‐discursive practices that spark emotional responses. GPs avoid negative isolation by crafting their work, but physical infrastructures can be altered too. New health centres in Finland have different spatial solutions, and some have even abandoned designated individual consulting rooms. If these kinds of multipurpose centres become the new norm of healthcare, it will shape the experiences of healthcare workers. However, the consequences are not always those originally hoped for, and people can adapt to institutional and spatial changes in surprising ways (VanHeuvelen 2019). In addition, the increasing amount of digital documentation work and the constant reforms that restructure healthcare services have not challenged isolation. In fact, the increasing digital work, ‘talking with the computer’ (Wrede et al. 2022, 85), might even reinforce isolation.

The concept of ‘good enough’ that arose from the data becomes a core issue in managing the workload in isolated consulting rooms. It is interesting also in relation to independence and collaboration. If the doctors choose to emphasise independence, doing a ‘good enough’ job requires coping individually with the problems at hand. In contrast, if collaborative work is possible, ‘good enough’ practice is the collective effort of the whole medical team. The different possibilities of crafting one's job and its links to other disadvantages in the medical profession would be an interesting topic for future research. Are collaborative crafting practices professionally more sustainable than promoting individual endurance? Who is able to craft their work the best? For example, according to previous research, female GPs face different expectations than their male counterparts and are expected to have a more patient‐centred practice and spend more time listening to patients (Mayson and Bardoel 2021). If female GPs have different or even fewer opportunities to craft their work, this will strengthen the gendered inequalities within the profession. The middle‐class masculinity and individual endurance have problematic effects on female junior doctors' opportunities to build their careers (Olakivi and Wrede 2019). Recently reported findings support the fact that gender‐based glass ceilings still prevail in Finnish medical work (Wrede et al. 2022).

This study shows how GPs make the institution more inhabitable for themselves—or themselves more suitable inhabitants of the institution. Whatever crafting activity they choose, GPs create and maintain the health centre, especially the consulting room, which serves as the arena for patient–doctor encounters. It is also the arena for accomplishing oneself as a professional in public primary healthcare. Thus, the lonely consulting room may not be professionally sustainable, especially for junior GPs starting their careers. For example, limited supervision can contest autonomy and enhance stratification among medical residents (Jenkins 2018), and unfulfilled career expectations can violate the psychological contract and career agency (Mitra et al. 2023). Although the Finnish primary care sector is in crisis and feared to collapse in the near future (Finnish Medical Journal, April 24, 2024), improving work situations by crafting can be a way of securing employees in the future. My analysis illustrates that enforcing collaborative and collegial or independent and autonomous doctoring are more rewarding ways of primary care doctoring than isolation and loneliness. Thus, acknowledging doctors' crafting practices and changing the material settings to enable collaboration would help improve the situation of (Finnish) primary care—which is suffering from a GP deficit—and keep doctors in their jobs.

## Author Contributions

**Inka Laisi:** conceptualization (lead), methodology (lead), formal analysis (lead), writing – original draft (lead), writing – review and editing (lead).

## Ethics Statement

This study adheres to the ethical standards set out by the Finnish National Board on Research Integrity (TENK). According to guidelines defined by TENK, an ethical review is only required in specific ethically challenging scenarios, none of which apply to this study.

## Conflicts of Interest

The author declares no conflicts of interest.

## Supporting information

Supporting Information S1

## Data Availability

The data that support the findings of this study are not publicly available due to privacy or ethical restrictions.
