# Supplementary material for: Crafting the Isolated Consulting Room: Practices of Independence and Collaboration of Finnish Primary Care GPs
Source: Sociol Health Illn. 2025 Jul 23;47(7):e70071. doi: 10.1111/1467-9566.70071 (PMC12285693; doi:10.1111/1467-9566.70071)
Supplement: Supplementary file 1 — Supporting Information S1 [file SHIL-47-0-s001.docx]

Crafting the isolated consulting room: Practices of independence and collaboration of Finnish primary care GPs

Appendix 1: Interview body

[Original in Finnish. Translated from Finnish to English by the author.]

**Work and study history and current employment**

*How long have you worked as a physician?*

*When did you start in your current position?*

*Do you also have other tasks or work in other positions?*

**Information about interviewee’s work and work organisation**

*Would you describe your main tasks, for example during one shift/workday?*

*Would you describe your work community? Who are the closest people you work with?*

*Are there any challenges in working with colleagues or other professionals? What kind? What about benefits? What kind?*

*Have you noticed any differences between doctors’ attitudes towards work? For example, between doctors in different career phases, between hospital and health centre doctors, men or women? What kind of differences?*

*How can you influence your work tasks, e.g. schedules?*

*Who are your supervisors/line-managers and what kind of things do they decide related to your work? Do you report to them?*

*What are the main challenges in your work? What increases your workload or pressure?*

*What is most rewarding in your work?*

**Medical community and views on doctors’ professional position in society**

*What does belonging to the medical community mean to you?*

*What do you think about doctors’ (or medical community’s) position in the society, and how do you see it changing in the future?*

**Vignettes**

Ask the interviewee to reflect on the vignette as the attending doctor.

Vignette 1:

A patient with an illness X comes to your office. Current Care Guidelines (Finnish national care guidelines) state that you should order treatments A. However, you have heard about a promising treatment B, with great outcomes. You would like to use it. Standard procedures would be cheaper. What do you do? Why? What kind of things affect your decision making?

Vignette 2:

A patient comes to your office. They found a diagnosis and medication on the internet and they came to ask for the prescription. The patient is right in the diagnosis, but you don’t think they need medication. The patient is clearly waiting for the prescription. What do you do? Why?

Vignette 3:

A patient comes to your office for the first time. They’ve had illness X for years, and the follow-up has been taken care of by a doctor you know. The doctor is now on vacation. You realise that the medication is not in line with the current care guidelines. What do you do? Why? What kind of things affect your decision making?

Vignette 4:

You work in a unit with an emergency room. Your contract obliges you to do on-call shifts in the emergency room. While working on call is an official duty of a doctor, on-call shifts must be equally distributed among the doctors, like The Finnish Medical Association states as well. However, there are colleagues who do not want to work on call and they have justified reasons. This creates problems in distributing the shifts. What do you think about the situation?

**Statements**

*We are asking about these statements, if you agree or disagree with them? Would you also reflect on your opinion, why do you think that way?*

Statement 1: The line-manager of a clinical physician does not have to be a medical doctor.

Statement 2: Current Care Guidelines restrict doctor’s decision making on patient care.

[Helping questions: have you encountered this kind of thinking among other doctors? Or can you find any arguments supporting this kind of thinking, even if you disagreed?]

Statement 3: Patients’ demands restrict doctor’s decision making on patient care.

[Helping questions: Helping questions: have you encountered this kind of thinking among other doctors? Or can you find any arguments supporting this kind of thinking, even if you disagreed?]

Statement 4: A doctor should act against the law, if the law is in conflict with the medical ethics. [If the interviewee cannot think of anything, mention for example, driving licenses, gun law, mental health issues]

Statement 5: Authorities of the state or the medical association should set minimum criteria on what is sufficient training (after graduating as medical doctor) for a clinical physician.

Statement 6: The Finnish Medical Association should regulate individual doctors more.

**Doctor autonomy**

*How do you understand the idea of doctor’s autonomy?*

*What about the autonomy of the medical community?*

*Can you think of any concrete examples in your own work, when you have experienced that your autonomy has been questioned? I’m thinking of situations, where you think patient care or security could have been compromised. Would you describe the situation and who were involved? How did it go?*

*Is doctor’s autonomy an important issue for you? Why / why not?*

*From which aspect of doctor’s autonomy would you not give up, under any circumstances?*

*Is there anything you would like to add, that I was not able to ask about?*
